# Counting-Based Effective Dimension and Discrete Regularizations

**DOI:** 10.3390/e25030482

**Published:** 2023-03-10

**Authors:** Ivan Horváth, Peter Markoš, Robert Mendris

**Affiliations:** 1 Nuclear Physics Institute CAS, 25068 Řež, Czech Republic; 2 Department of Physics and Astronomy, University of Kentucky, Lexington, KY 40506, USA; 3 Department of Experimental Physics, Faculty of Mathematics, Physics and Informatics, Comenius University in Bratislava, Mlynská Dolina 2, 842 28 Bratislava, Slovakia; 4 Department of Mathematics, Shawnee State University, Portsmouth, OH 45662, USA

**Keywords:** Minkowski dimension, effective counting dimension, effective number theory, effective support, effective description, minimal effective description, regularization, Anderson localization, lattice QCD

## Abstract

Fractal-like structures of varying complexity are common in nature, and measure-based dimensions (Minkowski, Hausdorff) supply their basic geometric characterization. However, at the level of fundamental dynamics, which is quantum, structure does not enter via geometric features of fixed sets but is encoded in probability distributions on associated spaces. The question then arises whether a robust notion of the fractal measure-based dimension exists for structures represented in this way. Starting from effective number theory, we construct all counting-based schemes to select effective supports on collections of objects with probabilities and associate the *effective counting dimension* (ECD) with each. We then show that the ECD is scheme-independent and, thus, a well-defined measure-based dimension whose meaning is analogous to the Minkowski dimension of fixed sets. In physics language, ECD characterizes probabilistic descriptions arising in a theory or model via discrete “regularization”. For example, our analysis makes recent surprising results on effective spatial dimensions in quantum chromodynamics and Anderson models well founded. We discuss how to assess the reliability of regularization removals in practice and perform such analysis in the context of 3d Anderson criticality.

## 1. Prologue

Consider the prototypical example of a fractal structure, the ternary Cantor set C⊂[0,1]. Evaluating its Minkowski dimension [1] involves the introduction of the regularization parameter a>0, namely the size of the elementary interval (“box”), and the use of ordinary counting to determine the number N(a) of such boxes required to cover C. The scaling of N(a) in the process of “regularization removal”, namely

(1) $$N\left(a\right)\;\propto \;a^{-d_{M}}\;\mathrm{for}\;a\to 0$$

then specifies the Minkowski dimension $d_{M}\left[C\right]$ ($=\;\log_{3}2$).

Assume now that, instead of a fixed set such as C, we are given a probability measure μ over the sample space [0,1] as a way to introduce structure on this interval. The ensuing probabilities make certain parts of the interval preferred over others, which is the probabilistic analogue of sharply selecting C in the case of fixed sets. Is it possible to characterize the probabilistic case by a robust fractal dimension with meaning akin to Minkowski?

Here we construct such a dimension and clarify in what sense it is unique. To convey the idea, consider a schematic analogue of the Minkowski prescription in the above example. As a **first step**, introduce a discrete regularization parameter a=1/N, where *N* now refers to the number of equally sized intervals forming a partition $I\;=\;\{I_{i}\;;\;i\;=\;1,2,\ldots ,N\}$ of [0,1]. With each $I_{i}$ associate the probability $p_{i}\;=\;\mu \left[I_{i}\right]$ to obtain the distribution $P\left(a\right)\;=\;\left(p_{1},\ldots ,p_{N(a)}\right)$. Thus, for each a∈{1,1/2,1/3,…}, we have a collection of Minkowski boxes which, however, come with probabilities. In the **second step**, assume we modify the ordinary counting N=N[I] of boxes to N=N[I,P] so that the probabilities are properly taken into account. In fact, N[I,P]=N[P] since *P* already carries the information on N[I]. Scaling of N upon the regularization removal, namely

(2) $$N\left[P\left(a\right)\right]\;\propto \;a^{-d_{\mathrm{UV}}}\;\mathrm{for}\;a\to 0$$

would then specify the dimension $d_{\mathrm{UV}}\;=\;d_{\mathrm{UV}}\left[\mu ,N\right]$ in analogy to (1). The subscript UV (ultraviolet) conveys that the regularization in question controls the structure at small distances. In this sense, $d_{M}$ is of course a UV construct as well.

The above plan for $d_{\mathrm{UV}}$ becomes a well-founded concept analogous to $d_{M}$ assuming that: (i) scheme N is additive like ordinary *N*; (ii) there is a notion of well-delineated effective support S=S[μ,N] induced by μ and specified by N, namely an analog of C; (iii) $d_{\mathrm{UV}}$ only depends on μ, i.e., $d_{\mathrm{UV}}\left[\mu ,N\right]\;=\;d_{\mathrm{UV}}\left[\mu \right]$ for all N satisfying (i) and (ii). Indeed, (i) makes $d_{\mathrm{UV}}$ measure-based in the same sense $d_{M}$ is, while (ii) allows for a structure induced probabilistically by μ to be described in the same way as structure of fixed sets. Part (iii) then guarantees that $d_{\mathrm{UV}}$ is robust (unique).

The notion of effective counting is clearly central to $d_{\mathrm{UV}}$. Its theoretical framework is the effective number theory (ENT) [2] which, among other things, determines all N satisfying (i). In this work, we give an affirmative status to (ii) and (iii) by developing the concept of the *effective counting dimension* (ECD), which is more general than $d_{\mathrm{UV}}$.

Before giving a self-contained account of the ECD, a few points are worth emphasizing. (a) The variety of fractal structures characterized by $d_{\mathrm{UV}}$ is much larger than those by $d_{M}$. For example, μ may describe the Cantor set with a non-uniform probability measure on it, for which $d_{M}$ is not applicable. (b) While probability/stochasticity play an important role in the fractal world [3], they usually enter differently than here. For example, a representative of a random Cantor set or an individual Brownian path arise via a random process, but such samples are still fixed sets. Our extension in these situations treats sample itself as a probability measure. (c) The above setup arises, e.g., in the quantum description of the natural world since quantum states encode probabilities of physical events. Here, it is common that the dynamics is in fact defined by regularization, be it UV or IR (system size L→∞). The input for computing $d_{\mathrm{UV}}$ or $d_{\mathrm{IR}}$ is then directly P(a) or P(L), rather than (usually unknown) μ. Such calculations of $d_{\mathrm{IR}}$ for Dirac eigenmodes in quantum chromodynamics [4] and for critical states of Anderson transitions [5] recently led to new geometric insights.

## 2. Qualitative Outline and Summary

In the study of physical and various model situations, we frequently need to describe or analyze some collection $O\;=\;\{o_{1},o_{2},\ldots \}$ of objects. The most basic quantitative analysis of *O* is to count its objects, namely to label it by a natural number *N*. However, the descriptive value of an ordinary count becomes limited when objects in a collection differ substantially. Indeed, consider $O_{•}$ containing the Sun, Proxima Centauri and $10^{10}$ individual grains of sand from Earth’s beaches. The ordinary count $N\;=\;2+10^{10}$ is clearly a poor characteristic of $O_{•}$ if the individual importance of its objects is judged by their masses.

The recent works [2,6,7] revisited counting with the aim of making it more informative in such situations. The resulting effective number theory studies possible ways to assign counts to collections of objects distinguished by an additive importance weight such as probability or mass. For example, with $O_{•}$ it associates an *intrinsic (minimal) effective count* by mass $N_{★}\;=\;2+2.0\times 10^{-16}$, leading to useful quantitative insight. (The textbook masses for the stars and the average mass of 4.5 mg for a grain of sand went into this calculation using Equation (5). Note that a specific normalization is involved in the prescription.)

The utility of ordinary counting in analyzing the natural world stems mainly from its *additivity*, which guarantees consistent bookkeeping for stable objects and leads to predictability: given disjoint collections $O_{1}$ and $O_{2}$ with labels $N_{1}$ and $N_{2}$, we can predict what the label of the combined collection $O_{1}\cup O_{2}$ would be ($N_{1}\;+\;N_{2}$) without actually performing the merger. Since merging and partitioning are at the heart of dealing with objects, counting became a theoretical tool: it is usually easier and faster to handle numbers than physical objects.

ENT requires any extension of counting to its effective form to be additive in order to preserve the above features [2]. Generalized additivity arises naturally if one views ordinary counting as a process carried out by a machine. Its “ready state” includes an empty list I=(), and its operation entails receiving objects from *O* sequentially. Each input causes *I* to update via I→I⊔(1), where ⊔ denotes concatenation, resulting in certain I=(1,1,…,1) upon exhausting all objects. The machine then calls its number function N=N[I] (length of a list) and outputs the label. This representation of ordinary counting makes it plain that the scheme is encoded by function N[I] and its additivity is expressed by the functional equation

(3) $$N\left[I_{1}⊔I_{2}\right]\;=\;N\left[I_{1}\right]+N\left[I_{2}\right],\;\forall \;I_{1}\;,\;I_{2}$$

In effective counting, each object *o* comes with a label specifying its own weight *w*. The associated machine initializes the weight list W=() and then sequentially inputs the objects. Upon each input, it scans the weight *w* and updates *W* via W→W⊔(w). After finishing the input, it calls its number function N=N[W] to obtain the ordinary count and uses it to rescale *W* into a canonical counting form $W\;\to \;C=(c_{1},c_{2},\ldots ,c_{N})$ satisfying $\sum_{i}c_{i}\;=\;N$. This rescaling is allowed since, like its ordinary prototype, effective counting is scale-invariant by construction. The machine then calls its effective number function N=N[C] and outputs the result. The additivity of the procedure is then expressed by the functional equation [2]

(4) $$N\left[C_{1}⊔C_{2}\right]=N\left[C_{1}\right]+N\left[C_{2}\right],\;\forall \;C_{1},\;C_{2}$$

It ensures that effective counts of disjoint collections with equal average weight per object add up upon merging.

Hence, in the same way that N=N[I] encodes ordinary counting, each N=N[C] obeying (4) and other necessary conditions [2] specifies a valid effective counting scheme. When used consistently in all situations, each offers bookkeeping and predictability features analogous to those of ordinary counting. ENT identifies all such effective schemes N. A key property of the resulting concept is that the scheme specified by [2]

(5) $$N_{★}\left[C\right]\;=\;\sum_{i=1}^{N}n_{★}\left(c_{i}\right),\;n_{★}\left(c\right)\;=\;\min\;\left\{c,1\right\}\;$$

satisfies $N_{★}\left[C\right]\;\leq \;N\left[C\right]\;\leq \;N\left[C\right]$ for all *C* and all N. Hence, the effective total prescribed by $N_{★}$ cannot be lowered by a change of counting scheme and is intrinsic to a collection. In fact, each collection of objects with additive weights is characterized by two key counting characteristics: the ordinary count *N* and the intrinsic effective count $N_{★}$.

In this work, we show that effective counting entails a unique notion of dimension. The associated setting involves an infinite sequence of collections $O_{k}$ with $N_{k}$ objects and an associated sequence $C_{k}\;=\;\left(c_{k,1},c_{k,2},\ldots ,c_{k,N_{k}}\right)$ of counting weights. Here $N_{k}$ is strictly increasing and, hence, $\lim_{k\to \infty }N_{k}\;=\;\infty$. The pair $O_{k}$, $C_{k}$ may specify e.g., an increasingly refined representation of a complex composite object or of a physical system with infinitely many parts. Following the standard physics language, we refer to it as “regularization” of the target k→∞ situation.

Assume that we fix a counting scheme N and associate with each weighted collection *O*, *C* its effective description $O_{s}\;=\;O_{s}\left[C,N\right]$, containing only the N[C] highest-weighted objects from *O*. To any regularization sequence $O_{k}$, $C_{k}$ this assigns a sequence of effective descriptions $O_{s,k}$ yielding the effective description of the target. Then, the idea of the *effective counting dimension* (ECD) is to convey how the abundance of objects in the effective description of the target scales with that in its full representation. In other words, ECD corresponds to Δ in

(6) $$N\left[C_{k}\right]\propto N{\left[C_{k}\right]}^{\;\Delta }\;\mathrm{for}\;k\to \infty ,\;\;0\leq \Delta \leq 1\;$$

However, it turns out that the above notion of the effective description (effective support) $O_{s}$ is only consistent for certain schemes N. Indeed, for N to delineate the support properly, a separation property formulated in Section 4 below has to be imposed. Formally, if N is the set of all schemes N, then only elements of its subset $N_{s}\;\subset N$ assign effective supports. We will show in Section 4 that $N_{s}$ is spanned by

(7) $$N_{\left(u\right)}\left[C\right]\;=\;\sum_{i=1}^{N}n_{\left(u\right)}\left(c_{i}\right),\;n_{\left(u\right)}\left(c\right)=\min\;\left\{c/u,1\right\}\;\;$$

where u∈(0,1]. Note that $N_{\left(1\right)}\;=\;N_{★}\in N_{s}$.

The above leads us to consider Δ=Δ[{C},N] with {C} a shorthand for the regularization sequence and $N\in N_{s}$. The minimal nature of $N_{★}$ implies that $\Delta_{★}\left[\left\{C\right\}\right]\equiv \Delta \left[\left\{C\right\},N_{★}\right]$ is the smallest possible ECD. However, ECD is in fact fully robust and does not depend on N at all. Indeed, in Section 5, we will show that

(8) $$\Delta_{★}\left[\left\{C\right\}\right]\;=\;\Delta \left[\left\{C\right\},N\;\right],\;\;\forall \;N\in N_{s}$$

Hence, ECD is a well-defined characteristic of the target specified by the regularization pair {O}, {C}. The use of additive counting makes it a measure-based effective dimension.

Before demonstrating the results (7) and (8), we wish to make a few remarks. (i) The fact that ECD does not require a metric allows for a large range of applications. Indeed, models in some areas (e.g., ecosystems and social sciences) often do not involve distances. (ii) The setups with metric frequently entail UV cutoff *a* (∝ shortest distance) and IR cutoff *L* (∝ longest distance). Sequence {C} can then facilitate their removals: $C_{k}$ may be associated e.g., with $a_{k}\;\to \;0$ at fixed *L* or with $L_{k}\;\to \;\infty$ at fixed *a*. Defining the nominal dimensions via $N\left[C_{k}\right]\;\propto a_{k}^{-D_{\mathrm{UV}}\left(L\right)}$ and $N\left[C_{k}\right]\;\propto L_{k}^{D_{\mathrm{IR}}\left(a\right)}$ for the UV and IR cases, their effective counterparts are [4]

(9) $$N_{★}\left[C_{k}\right]\propto a_{k}^{-d_{\mathrm{UV}}\left(L\right)},\;\;N_{★}\left[C_{k}\right]\propto L_{k}^{d_{\mathrm{IR}}\left(a\right)}\;$$

If $\Delta_{\mathrm{UV}}$, $\Delta_{\mathrm{IR}}$ denote their associated ECDs, then

(10) $$d_{\mathrm{UV}}=\Delta_{\mathrm{UV}}\;D_{\mathrm{UV}},\;\;d_{\mathrm{IR}}=\Delta_{\mathrm{IR}}\;D_{\mathrm{IR}}\;$$

Dimension $d_{\mathrm{IR}}$ was recently calculated in QCD [4] and in the Anderson models [5]. (iii) The meaning of $d_{\mathrm{UV}}$ is fully analogous to the Minkowski (box-counting) dimension of fixed sets. In fact, for {O} that UV-regularizes a bounded region in $R^{D}$, $d_{\mathrm{UV}}$ is exactly the Minkowski dimension of $\left\{O_{s}\right\}$ treated as a fixed set. The demonstrated uniqueness of the ECD suggests that measure-based dimensions are meaningful even for geometric figures emerging effectively, e.g., from probabilities. (iv) We refer to $O_{s}\;=\;O_{s}\left[O,C,N\right]$, $N\;\in \;N_{s}$, as both the support and the description of *O*. The latter is more suitable in situations involving information and complexity. Our analysis implies the existence of a well-defined *minimal effective description* $O_{★}\left[O,C\right]$, namely $O_{s}\left[O,C,N_{★}\right]$, which may find uses in these contexts.

## 3. Effective Counting Schemes

Our starting point is ENT [2], which determines the set N of all effective counting schemes $N\;=\;N\left[C\right]\;=\;N\left(c_{1},\ldots ,c_{N}\right)$. Apart from the additivity (4), symmetry, continuity, and boundary conditions, the axiomatic definition of N also ensures that increasing the cumulation of weights in *C* does not increase the effective number. This monotonicity is expressed by

(11) $$N\left(\ldots c_{i}+\epsilon \ldots c_{j}-\epsilon \ldots \right)\;\leq \;N\left(\ldots c_{i}\ldots c_{j}\ldots \right)\;$$

for each $c_{i}\;\geq \;c_{j}$ and $0\;\leq \;\epsilon \;\leq \;c_{j}$. The resulting N consists of additively separable functions $N\left(c_{1},\ldots ,c_{N}\right)\;=\;\sum_{i=1}^{N}n\left(c_{i}\right)$, such that the *counting function* n=n(c), c∈[0,∞), is

(12) $$\begin{array}{cc} \; & (i)\;\mathrm{continuous}\\ \; & (ii)\;\mathrm{concave} \end{array}\;\;\begin{array}{cc} \; & (iii)\;\;n(0)=0\\ \; & (iv)\;\;n(c)=1\;\;\mathrm{for}\;\;c\geq 1 \end{array}$$

The representation of N∈N by n satisfying (12) is unique.

## 4. Effective Supports

We assume from now on that the order of objects in $O=\{o_{1},\ldots ,o_{N}\}$ is set by their relevance in *C*, i.e., that $c_{1}\geq c_{2}\geq \ldots \geq c_{N}$. Given a counting scheme N, we collect the first N[C] objects to form the intended effective support (effective description) $O_{s}$ of *O*. Since N is real-valued, we represent $O_{s}$ as

(13) $$O\;=\;\left\{o_{1},\ldots ,o_{N}\right\}\;⟶\;\;O_{s}\left[O,C\right]=\left\{o_{1},\ldots ,o_{J},f\right\}$$

where *J* is the ceiling of N[C] and N[C]=(J−1)+f. Hence, 0<f≤1 is the fraction of $o_{J}$ included in $O_{s}$.

The rationale for effective support so-conceived is clear: $O_{s}$ is a subcollection of the most relevant elements from *O* that behaves under the ordinary counting measure in the same way as *O* under the effective one. Indeed, the additivity (4) translates into (dependence on N, $C_{1}$, $C_{2}$ is implicit)

(14) $$N\left[O_{s}\left[O_{1}⊔O_{2}\right]\right]=N\left[O_{s}\left[O_{1}\right]\right]+N\left[O_{s}\left[O_{2}\right]\right]$$

where an obvious real-valued extension of N[…] to collections with a fractional last element was made.

However, ENT axioms only deal with counting, and their compatibility with the above notion of effective support needs to be examined. Effective numbers are crucially shaped by additivity (4) and monotonicity (11). With additivity being the basis for $O_{s}$ via (14), it is the monotonicity that requires attention. To that end, consider the operation on the left-hand side of (11), involving the last object included in $O_{s}$ (i.e., $o_{J}$) and the first object fully left out ($o_{J+1}$). Since $o_{J}$ gains relevance at the expense of $o_{J+1}$, its presence in $O_{s}$ (measured by *f*) cannot decrease. This *separation property* is expressed by

(15) $$N\left(\ldots c_{J}+\epsilon ,c_{J+1}-\epsilon \ldots \right)\;\geq \;N\left(\ldots c_{J},c_{J+1}\ldots \right)\;\;$$

for all *C* such that $c_{J}\;>\;c_{J+1}\;>\;0$ and all sufficiently small ϵ>0. Ensuring a meaningful split of the effective support from the rest, (15) has to hold in order to define $O_{s}$ consistently. Note that J=ceil(N[C]) depends on both N and *C*.

We now show that the only counting schemes N compatible with (15) are specified by Equation (7). To start, note that, in order to make the separation property compatible with the monotonicity (11), we have to impose the equality sign in (15). In terms of counting function n of N, we have

(16) $$n\left(c_{J}+\epsilon \right)+n\left(c_{J+1}-\epsilon \right)=n\left(c_{J}\right)+n\left(c_{J+1}\right)$$

for all *C* with $c_{J}\;>\;c_{J+1}\;>\;0$ and all sufficiently small ϵ>0.

Note next that the properties (12) of n imply the existence of 0<u≤1 such that n(c)=1 for all c≥u, and 0<n(c)<1 for all 0<c<u. Given this u=u[n], the separation operation in (16) cannot be performed when $c_{J}\;\geq \;u$. Indeed, since each $n(c_{j})$ with j≤J contributes unity to N[C], we have $J\;=\;\mathrm{ceil}(J\;+\;\sum_{i=J+1}^{N}c_{i})$, leading to $c_{J+1}\;=\;0$. Hence, it is sufficient to consider (16) for *C* with $u\;>\;c_{J}\;>\;c_{J+1}\;>\;0$. In this form, it is readily satisfied by $n_{\left(u\right)}$ of Equation (7) due to its linearity on [0,u]. Consequently, $N_{\left(u\right)}\;\in N_{s}$.

However, all other n featuring the same *u* violate (16). To show that, consider N=3 vectors C=(3−y−x,y,x) with 0<x<y<u and J=2. The definition of *J*, namely J−1<N[C]≤J, demands that n(x)+n(y)≤1. We will specify the *x*, *y* that satisfy this, as well as $\epsilon_{0}\;>\;0$ such that n(x−ϵ)+n(y+ϵ)<n(x)+n(y) for all $0\;<\;\epsilon \;<\;\epsilon_{0}$, thus failing to satisfy (16). Since $n_{\left(u\right)}$ produces an equality in this relation, we can proceed using $g\left(x\right)\;=\;n\left(x\right)\;-\;n_{\left(u\right)}\left(x\right)$ and

(17) $$g\left(x-\epsilon \right)+g\left(y+\epsilon \right)\;<g\left(x\right)+g\left(y\right),\;\;0<\epsilon <\epsilon_{0}$$

From the properties (12) of n, $n_{\left(u\right)}$ and the explicit form of $n_{\left(u\right)}$, it follows that g(x) is a continuous function satisfying:

(a) g(0)=g(u)=0; (b) g(x)>0 for 0<x<u; (c) there are $0\;<\;x_{0}\;\leq \;y_{0}\;<\;u$ such that g(x) is increasing on $\left[0,x_{0}\right]$ and decreasing on $\left[y_{0},u\right]$. Hence, any $0\;<\;x\leq x_{0}$, $y_{0}\;<\;y\;<\;u$ and $0\;<\;\epsilon_{0}\;\leq \;\min\left\{x,u-y\right\}$ form a triple satisfying (17). Finally, for any *y* chosen as above, we can select sufficiently small x>0 such that n(x)<1−n(y) due to n(0)=0 and continuity. Hence, all schemes N based on $n\neq n_{\left(u\right)}$ fail to satisfy (15), and $N_{s}$ is spanned by $N_{\left(u\right)}$.

## 5. Uniqueness of ECD

It is now straightforward to show (8). Consider a pair $N_{★}$, $N_{\left(u\right)}$ for arbitrary, but fixed 0<u≤1. We will compare their counting functions $n_{★}\left(c\right)$ and $n_{\left(u\right)}\left(c\right)$ on the following partition of their domain: (i) 0≤c≤u. Here, $n_{★}\left(c\right)\;=\;c$ and $n_{\left(u\right)}\left(c\right)=c/u$, and hence, $n_{\left(u\right)}\left(c\right)=n_{★}\left(c\right)/u$. (ii) u<c<1. Here, $n_{★}\left(c\right)=c$ and $n_{\left(u\right)}\left(c\right)=1$, and so, $n_{\left(u\right)}\left(c\right)=n_{★}\left(c\right)/c<n_{★}\left(c\right)/u$. (iii) c≥1. Here, $n_{★}\left(c\right)=n_{\left(u\right)}\left(c\right)=1$, and so, $n_{\left(u\right)}\left(c\right)\leq n_{★}\left(c\right)/u$.

Taken together, this yields $n_{★}\left(c\right)\leq n_{\left(u\right)}\left(c\right)\leq n_{★}\left(c\right)/u$ for any c≥0, as well as

(18) $$N_{★}\left[C\right]\leq N_{\left(u\right)}\left[C\right]\leq \frac{1}{u}\;N_{★}\left[C\right],\;\forall \;C\;,\;\forall \;u\;\in \;\left(0,1\right]$$

Now, consider a regularization sequence {C} such that its ECD (6) associated with $N_{★}$ (i.e., $\Delta_{★}$) exists. From (18), it follows that the power governing the growth of $N_{\left(u\right)}\left[C_{k}\right]$ with $N_{k}$ also exists and is equal to $\Delta_{★}$, as claimed in (8). Hence, the concept of the effective counting dimension associated with {C} is well-defined (unique).

## 6. Generality of ECD

The general context we associated with the ECD, namely that of arbitrary collections *O* of objects, may raise questions about using the term “dimension”. Indeed, its intuitive notion is frequently reserved for less general setups and, in particular, for those involving a metric (see Comment (i) in Section 2). We thus elaborate more on the underlying rationale.

In line with the usual practice, we aimed at minimal conditions under which the notion of the ECD is applicable. Such a minimal setup turns out to be a sequence $\left(O_{k},C_{k}\right)$ of objects and associated additive weights. This arises due to the fact that, while ordinary counting does not give any structure to this most bare of settings, the effective counting does. In fact, the most-relevant consequence of the present analysis is that ECD provides the robust and well-founded quantitative characteristic of this structure.

To illustrate the reasoning, consider an extreme example of a sequence where $\left(O_{1},C_{1}\right)$ involves apples, $\left(O_{2},C_{2}\right)$ potatoes, $\left(O_{3},C_{3}\right)$ apples again, $\left(O_{4},C_{4}\right)$ peanuts, and so on. While a casual observer presented with the sequence may be puzzled by its meaning, it may have a clear rationale for a fertilizer company, which generated it as part of their efficiency analysis. The associated ECD has the same nominal meaning for both (it specifies how the effective number of objects scales with their ordinary number), but the fertilizer company will find it natural to call it a dimension. After all, at the heart of ordinary measure-based dimensions is the scaling of the measure, and they are dealing with scaling of their own measure represented by the effective count. The casual observer, from whom the meaning of effective count is hidden, may object.

In the next section, we will discuss an example of ECD stability study using critical wave functions of 3D Anderson transitions. This involves sequences labeled by size *L* of the system which, similarly to the example above, may seem like sequences of independent distinct objects. However, they have common origin in probability distributions of wave functions generated by quantum dynamics of the Anderson model, which makes the sequences meaningful. Moreover, the objects are actually elements of physical space in this case, which allows us to interpret the ECD as an effective dimension of space occupied by the Anderson electron.

## 7. Anderson Criticality

The results of [2] and the present work suggest that using $N_{★}$ alone suffices for many effective counting analyses. However, even then, additional input from other schemes in N may be informative. For example, it can be used to assess the reliability of regularization removals. Indeed, $N_{s}$ is spanned by schemes $N_{\left(u\right)}$ and we showed that the associated Δ(u) is constant. However, carrying out the k→∞ extrapolation in practice can be affected by large systematic errors if available collections are not sufficiently large to achieve scaling in (6). The computed Δ(u) is then expected to vary significantly. On the other hand, when approximate scaling is in place, the degree of non-constant behavior can be used to judge the level of systematic errors.

To explain, note first that possible effective supports $O_{s}\;=\;O_{s}\left(u\right)$ of *O* contain populations of $N_{\left(u\right)}$ objects that decrease with increasing *u*. Hence, for given $O\;=\;\{O_{k_{1}},O_{k_{2}},\ldots ,O_{k_{M}}\}$ used in regularization removal, function Δ(u,O) is also expected to be decreasing. At the same time, *u* can be lowered to make the fraction of objects in the effective support arbitrarily close to one, and there is a guaranteed over-representation of the scaling population at sufficiently small *u*. In fact, it is expected that $\lim_{u\to 0}\Delta \left(u,O\right)\;\approx \;1$ for generic O, regardless of the true ECD. The signature of O suitable for regularization removal is the existence of a “scaling window” in *u*, where Δ(u,O) changes slowly. The value $\Delta (u_{0},O)$ at the point $u_{0}$ of slowest change is expected to produce the most-reliable estimate of ECD from O. The change of Δ(u,O) within the window sets an approximate scale of systematic error.

We now apply this general strategy to the recent calculation [5] of spatial effective dimensions $d_{\mathrm{IR}}$ at Anderson transitions [8,9,10] in three dimensions ($d_{\mathrm{IR}}\;=\;3\Delta_{\mathrm{IR}}$). We will focus on the 3d Anderson model in the orthogonal class, defined on the ${\left(L/a\right)}^{3}$ cubic lattice with sites labeled by $r\;=\;(x_{1},x_{2},x_{3})$ and periodic boundary conditions. The model is diagonal in spin, and it is thus sufficient to consider one-component fermionic operators $c_{r}$. Denoting by $\epsilon_{r}$ the on-site random energies chosen from a box distribution in the range [−W/2,+W/2], the Hamiltonian is [8]

(19) $$H\;=\;\sum_{r}\epsilon_{r}\;c_{r}^{†}\;c_{r}\;+\;\sum_{r,j}c_{r}^{†}\;c_{r-e_{j}}+h.c.$$

Here, $e_{j}$ (j=1,2,3) are unit lattice vectors. For energy E=0, there is a critical point at $W\;=\;W_{c}\;=\;16.543\left(2\right)$ [11] separating extended states at $W\;<\;W_{c}$ from exponentially localized ones at $W\;>\;W_{c}$.

Objects $o_{i}$ involved in the calculation of $d_{\mathrm{IR}}\;=\;d_{\mathrm{IR}}\left(E,W\right)$ are elementary cubes of space at positions $r_{i}$, with weights specified by wave function ψ via $w_{i}\;=\;p_{i}\;=\;\psi^{+}\psi \left(r_{i}\right)$. Collection *O* forms the space occupied by the system with volume $V\;=\;N\left[O\right]a^{3}\;=\;L^{3}$. Electron in state ψ is effectively present in a subregion $O_{★}\left[\psi \right]$ of volume $V_{\mathrm{eff}}\;=\;N_{★}\left[\psi \right]a^{3}$. Dimension $d_{\mathrm{IR}}$ gauges the asymptotic response of $V_{\mathrm{eff}}$ to increasing *L*. The model involves averaging over disorder $\left\{\epsilon_{r}\right\}$, and hence, $N_{★}\;\to \;\langle \;N_{★}\rangle$ in the definition (9). The critical effective dimension $d_{\mathrm{IR}}\left(0,W_{c}\right)\;\approx \;8/3$ was found for (19) and models in three other universality classes [5]. This commonality was expressed by super-universal value $d_{\mathrm{IR}}^{\mathrm{su}}\;=\;2.665\left(3\right)$ with the quoted uncertainty including the spread over classes.

We generated a new set of data for system (19) at the critical point $\left(0,W_{c}\right)$, producing O including 26 systems with sizes in the range 22≤L/a≤160. The JADAMILU library [12] was used for matrix diagonalization. We then strictly followed the analysis procedure of [5] and obtained $d_{\mathrm{IR}}\left(O\right)=2.6654\left(11\right)$, consistent with the original estimate. However, in the present calculation of critical eigenmodes, we recorded $N_{\left(u\right)}$ for 20 equidistant values of *u* starting with u=0.05, rather than just $N_{★}\;=\;N_{\left(1\right)}$. This allowed us to perform the proposed stability analysis utilizing $N_{s}$.

The latter is most efficiently performed by computing $\delta \left(u,O\right)\;=\;d_{\mathrm{IR}}\left(u,O\right)\;-\;d_{\mathrm{IR}}\left(1,O\right)$, which can be extracted directly from the raw data without any intermediate steps. Indeed, the large *L* behavior of ${\langle N_{\left(u\right)}\rangle }_{L}/{\langle N_{\left(1\right)}\rangle }_{L}$ is governed by power δ(u). Moreover, relation $d_{\mathrm{IR}}\left(u\right)\;=\;d_{\mathrm{IR}}$ is replaced by the definite δ(u)=0. While still featuring the expected decreasing behavior and slow variation in the scaling window, the size of δ(u,O) directly conveys the scale of systematic errors. Note that, since the above ratio defining δ(u) involves correlated data in the way we performed the calculation, the Jackknife procedure was used to estimate its error in the analysis described below.

**Figure 1:**
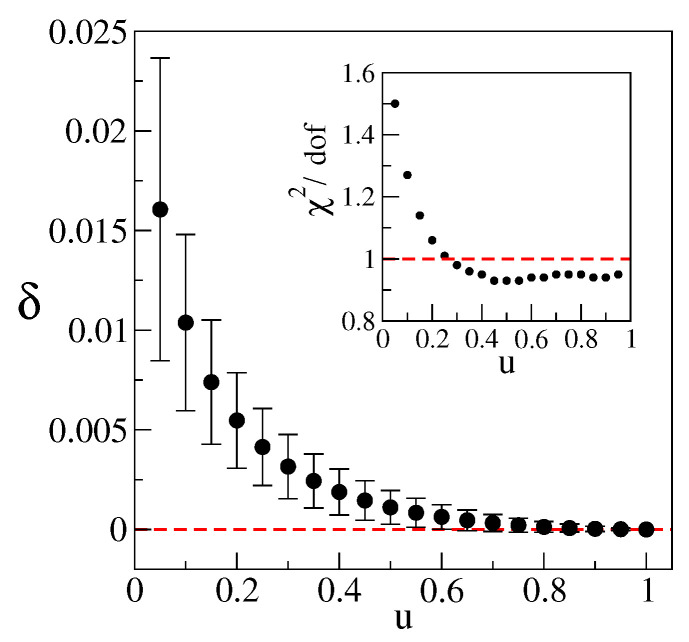
Deviation $\delta \left(u,O\right)\;=\;d_{\mathrm{IR}}\left(u,O\right)\;-\;d_{\mathrm{IR}}\left(1,O\right)$ obtained via 2-power fit as described in the text. The inset shows $\chi^{2}$ per degree of freedom (dof) for the fits involved.

To extract δ(u,O) from the data in an unbiased way, we included it as a parameter in general two-power fits of the above ratio in the form $c_{1}L^{\delta }+c_{2}L^{-\beta }$. The role of the second power is to absorb finite-volume effects, and its presence resulted in very stable results. Unconstrained two-power fits were mainly afforded by our extensive statistics (30K–100K of disorder realizations). We proceeded by finding the smallest size $L_{\min}$ in O, such that the fit in the range $L_{\min}/a\;\leq \;L/a\;\leq \;160$ yielded $\chi^{2}/\mathrm{dof}\;<\;1$ for u=0.95 data. The resulting $L_{\min}=30a$ was then fixed for fits at all *u*, leading to δ(u,O) shown in Figure 1. The respective $\chi^{2}/\mathrm{dof}$ are shown in the inset.

The resulting δ(u,O) is indicative of O suitable for regularization removal. Indeed, populations associated with the window 0.75≤u≤1 scale essentially in sync. The slowest change occurs at $u_{0}\;=\;1$, suggesting that the quoted result for $d_{\mathrm{IR}}$, which is based on $N_{★}$, is nominally the most-reliable for this O. Note that, according to Equation (18), effective support at u=1/2 is up to twice as abundant as the minimal one. The associated δ(1/2,O)≈0.002 offers a convenient canonical benchmark for the level of systematic error. Given the position of the scaling window and its degree of stability, it is likely an upper bound in this case. These findings suggest that $\approx \;10^{-3}$ is the scale of statistical, as well as systematic error associated with the calculation of $d_{\mathrm{IR}}$ in [5].

## Data Availability

The numerical data presented here is available upon request.

## References

[1] Falconer K. (2014). Fractal Geometry: Mathematical Foundations and Applications.

[2] Horváth I., Mendris R. (2020). Effective Number Theory: Counting the Identities of a Quantum State. Entropy.

[3] Bishop C.J., Peres Y. (2016). Fractals in Probability and Analysis.

[4] Alexandru A., Horváth I. (2021). Unusual Features of QCD Low-Energy Modes in the Infrared Phase. Phys. Rev. Lett..

[5] Horváth I., Markoš P. (2022). Super-Universality in Anderson Localization. Phys. Rev. Lett..

[6] Horváth I. (2021). The Measure Aspect of Quantum Uncertainty, of Entanglement, and the Associated Entropies. Quantum Rep..

[7] Horváth I., Mendris R. (2019). A Different Angle on Quantum Uncertainty (Measure Angle). Multidiscip. Digit. Publ. Inst. Proc..

[8] Anderson P.W. (1958). Absence of diffusion in certain random lattices. Phys. Rev..

[9] Evers F., Mirlin A.D. (2008). Anderson transitions. Rev. Mod. Phys..

[10] MacKinnon A., Kramer B. (1981). One-parameter scaling of localization length and conductance in disordered systems. Phys. Rev. Lett..

[11] Slevin K., Ohtsuki T. (2018). Critical exponent of the Anderson transition using massively parallel supercomputing. J. Phys. Soc. Jpn..

[12] Bollhöfer M., Notay Y. (2007). JADAMILU: A software code for computing selected eigenvalues of large sparse symmetric matrices. Comput. Phys. Commun..

